# The association between secondhand smoke exposure and growth outcomes of children: A systematic literature review

**DOI:** 10.18332/tid/117958

**Published:** 2020-03-03

**Authors:** Siti R. Nadhiroh, Kusharisupeni Djokosujono, Diah M. Utari

**Affiliations:** 1 Department of Nutrition, Faculty of Public Health, Universitas Airlangga, Surabaya, Indonesia; 2 Department of Nutrition, Faculty of Public Health, Universitas Indonesia, Depok, Indonesia

**Keywords:** secondhand smoke, children, growth, prenatal, postnatal

## Abstract

**INTRODUCTION:**

The strong relation between maternal smoking and maternal secondhand smoke (SHS) exposure and the growth of newborn infants has been proven. However, the effect of SHS on growth outcomes of older children is not well defined. Through a systematic literature review, we sought to determine whether a relationship exists between SHS exposure and growth outcomes of children up to 8 years of age.

**METHODS:**

A systematic review was performed, including articles published between 2004–2019, related to SHS exposure (prenatal and postnatal) and children’s growth (weight, length/height, and head circumference). The relevant articles were identified from Science Direct, ProQuest, Sage Publication, Scopus, Wiley Online Library, CINAHL Plus with Full Text (via EBSCOhost) and Google search.

**RESULTS:**

Seventeen articles were identified, of which three categories of growth measurements were extracted, comprising weight (weight, WAZ, WHZ, and BMI), height (height/length and HAZ) and head circumference. SHS exposure both pre or postnatally was inversely associated with weight (deficit in weight, risk of underweight, risk of wasting) and height (lower length and risk of stunting) and elevated BMI of children. Furthermore, prenatal SHS exposure was associated with a lower head circumference.

**CONCLUSIONS:**

The current review identified that exposure to SHS may be associated with adverse growth outcomes in children. It is crucial that active smokers, specifically those who live with children or with a pregnant partner, are made aware of the potential effects of SHS exposure on non-smokers. Further assessment of the association between exposure to SHS and other growth outcomes in other age groups is needed.

## INTRODUCTION

More than a third of the global population are passive smokers and regularly exposed to the dangerous effects of tobacco smoke. Smoke exposure is responsible for approximately 0.6 million deaths annually and approximately 1% of global disease around the world^1^. The result of a study across 192 countries showed that 40% of children were exposed to secondhand smoke (SHS)^2^ and 36% were exposed to SHS in utero^3^. This makes the implications of exposure a potentially significant public health problem.

Early childhood (usually defined as a newborn baby until the age of 8 years) is the phase of incredible growth in several aspects: physical, cognitive, social-emotional, and language skills^4,5^. During the early years, the brain develops quickly and has a high capacity for change, with the foundation set for health and wellbeing throughout life. Therefore, this period is critical. Protecting children from threat, including secondhand smoke exposure, is part of nurturing care that is sensitive to children’s health and nutrition needs^5^.

The existing studies showed that SHS exposure has a strong relation with low birth weight^6-8^, premature birth^9^, shorter baby length^10^, higher risk of fetal death, congenital defects^11^, and childhood obesity^12^. To date, limited information has been collated to illustrate the association between SHS exposure in non-smoking mothers during pregnancy and/or in children during postnatal life and the growth of children. Two review studies examined the association between SHS exposure and children growth outcomes, focusing on tobacco use of the mother during pregnancy^13,14^. Two other review articles explored the impact of SHS exposure on non-smoking pregnant women on anthropometric growth of children, focusing on the newborn baby^15,16^.

The objective of this systematic literature review was therefore to determine whether SHS exposure was associated with growth outcomes in children up to 8 years of age.

## METHODS

### Search strategy

We identified the eligible literature through a systematic search in 7 electronic databases: Science Direct, ProQuest, Sage Publication, Scopus, Wiley Online Library, CINAHL Plus with Full Text (via EBSCOhost) and Google search, without time-window restriction. The search used a combination of keywords from SHS (tobacco, tobacco smoke, environmental tobacco smoke, passive smoking, and secondhand smoke) and growth (anthropometric, growth, weight, length, and head circumference). After the abstracts were retrieved and screened, we evaluated the full text of the articles that related to SHS exposure and children’s growth. The additional articles were searched using the bibliography of the selected articles. The literature search was completed in June 2019.

### Inclusion and exclusion criteria

To be eligible for inclusion, the article must present the data from an observational study that includes both a measure of SHS exposure (pre or postnatal) and that at least one of the research objectives is measuring the children’s growth through anthropometric measurements. The anthropometry indices were weight, height, head circumference, weight-for-age z-score (WAZ), length or height-for-age z-score (HAZ), weight-for-length or weight-for-height z-score (WHZ), head circumference z-score (HCZ), body mass index-for-age z-score (BMIZ) or BMI-for-age percentile, and BMI or Kaup index. BMI and Kaup indices divide weight by the square of the height (kg/m^2^)^17,18^. Underweight (< -2 SD WAZ), stunting (< -2 SD HAZ), wasting (< -2 SD WHZ and BMIZ) and overweight (> +2 SD WHZ and BMIZ; ≥85 percentile BMI-for-age) are used to measure nutritional imbalance resulting in malnutrition (assessed from underweight, wasting, and stunting) and overweight^19-21^. Also, Z scores < -3 SD for WHZ, WAZ or HAZ were considered as severely wasted, severely underweight, or severely stunted^22^.

Retrieved articles were excluded if the exposure and outcome variables were not defined clearly or if the association of the growth outcome with SHS exposure could not be determined independently of other toxins such as air pollution or illicit drug exposure in utero, due to these factors being combined into one variable. This paper focused on SHS exposure on children aged <8 years. Therefore, if an article only included children aged ≥8 years, the article was excluded.

In addition, if no statistical evidence relevant to our research question was presented (e.g. data not shown) or were not original research articles, these were also excluded^23^. As the effect of maternal smoking in the prenatal period on the growth of the offspring has been reviewed thoroughly elsewhere^13,14^, the aim of the present review was to emphasize SHS exposure from other people smoking (i.e. paternal smoking). Therefore, articles that had data only on maternal smoking in pregnancy were excluded from the present systematic literature review.

### Data quality assessment

The quality of the studies was appraised using a scale adapted from the Newcastle/Ottawa Scale (NOS) (the appraisal standard of NOS is presented in the Supplementary file). Each study was assessed using the point system based on the NOS. One point was added when a study included relevant information that could be related to the NOS. Eight items in cohort studies and five items in cross-sectional studies that could be related to the NOS were identified. Hence, cross-sectional studies assigned 5, 4, 3, or 0–2 points, were assessed as ‘very good’, ‘good’, ‘satisfactory’ or ‘unsatisfactory’, respectively. Also, cohort studies with 7–8, 5–6, 4, or 0–3 points, were classified as ‘very good’, ‘good’, ‘satisfactory’ or ‘unsatisfactory’, respectively. Unsatisfactory studies were excluded^24,25^.

### Data extraction

Any issues that occurred are discussed below for each article. For all articles, the following data were independently extracted: year of publication, study design, participant sampling, country, number of participants, mean participant age, participant gender, percentage of participants exposed to SHS, SHS measurement, growth measurement, covariates included in the analysis, and the study outcome.

## RESULTS

**Figure 1:**
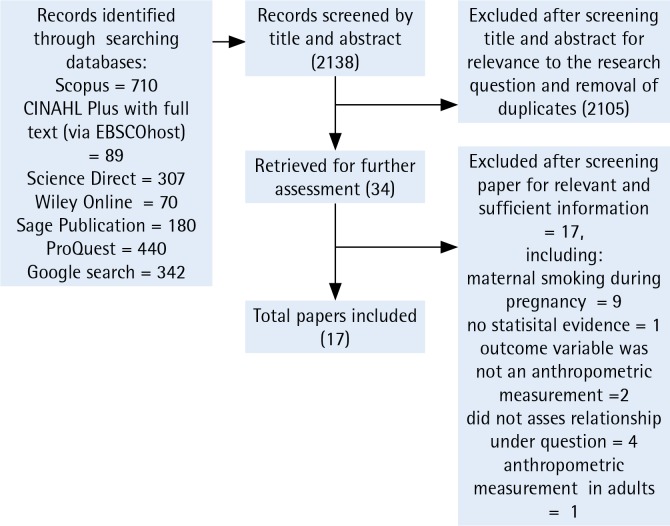
Flowchart for article selection

The literature search identified 2138 records, of which 2105 were excluded after screening title and abstract for relevance to the research question and removal of duplicates. Using the inclusion and exclusion criteria, we selected 34 full texts for further assessment. A total of 17 studies were excluded after screening for relevant and sufficient information, including maternal smoking during pregnancy only, no statistical evidence, not using an anthropometric measure, not assessing relationship under question, and anthropometric measurement in adults only. In total, 17 studies were included in our final systematic review (Figure 1).

The articles were published between 2004–2019, eight were cross-sectional^21,26-32^, and the other nine studies were prospective studies^17,18,33-39^. The age range included in this review was from 0 to 7 years old. Nine studies were conducted in Asian countries, two in the USA, four in Europe, and the last two^29,32^ were conducted in multi-countries (18 and 7 countries, respectively). Four articles used a biomarker for SHS exposure assessment, including cotinine serum^17^, plasma cotinine^18^, and urinary cotinine^34,37^. Meanwhile, the other articles used an interview or self-report through a questionnaire, from parents or caregivers, to estimate the SHS exposure. The articles are presented in two sections: growth measurements in children exposed to prenatal SHS (n=5; Table 1), and growth measurements in children exposed to postnatal SHS (n=12; Table 2).

**Table 1:** Growth measurements in children who had been exposed to SHS during the prenatal period

*Authors (year)*	*Methodology, sample and location*	*N*	*Population/sample characteristics*	*Measurement of SHS exposure*	*Measures of anthropometric functioning*	*Confounders measured*	*Outcomes*
Fenercioglu et al.^33^ (2009)	Prospective cohort, Turkey	159	Infant assessed at age 0, 3 and 6 months; 50.3% female;35.8% exposed to SHS	Self-report by mother. Exposed to SHS if a household member smoked ≥10 cigarettes/day inside the house	Weight, length, HC	Maternal education, economic status of family, parity, age, pre-pregnancy weight and height, paternal height	SHS exposure associated with deficit in children weight (mean= -378.16; 95% CI : -708.21, -48.10; p<0.01), length (mean= -2.26; 95% CI: -3.61, -0.91; p<0.01) and head circumference (mean= -1.17; 95% CI: -1.77, -0.56; p<0.01) at 3rd month compared to children not exposed to SHS
Braun et al.^17^(2010)	Prospective birth cohort, USA	292	Infant assessed at birth, 4 weeks, and 1, 2 and 3 years; % female (not given); 51% exposed to SHS	Interview with mother and prenatal serum cotinine (exposed vs not exposed)	BMIZ	Socio-demographic (maternal age, race, education, marital status and household income), perinatal variables (maternal depression, maternal BMI and parity) and childhood nutrition	SHS exposure associated higher BMI at 2 years (mean difference= 0.3; 95% CI: -0.1, 0.7) and 3 years (mean difference= 0.4; 95% CI: 0.0, 0.8) compared with unexposed children (p-value not reported)
Braimoh et al.^18^ (2017)	Hokkaido large-scale cohort, Japan	1356	Infant assessed at birth, 1.5 and 3 years; 50.1% female; 58.9% exposed to SHS	Maternal plasma cotinine (exposed vs not exposed)	Kaup index used by dividing the weight by the square of the height (kg/m^2^)	maternal age, height, weight before pregnancy, annual household income, maternal education level, infant gender, gestational age, maternal and partners’ smoking status (yes/no) at 1, 2 and 4 years after delivery; and breast feeding	SHS exposure associated with smaller Kaup index gain from birth up to 3 years of children born to passive smokers than in those born to non-passive smokers (−0.34 kg/m^2^; 95% CI: −0.67, −0.01; p<0.05)
Robinson et al.^34^ (2016)	Spanish INMA prospective birth cohort, Spain	1866	Infant assessed at 4 years; % female (not given); 29.6% exposed to SHS	Interview with mother and prenatal urinary cotinine (exposed vs not exposed)	BMIZ	Socioeconomic status, maternal country of origin, maternal age, maternal BMI, breastfeeding, and child physical and sedentary activity at 4 years, paternal BMI, maternal physical activity and alcohol consumption, maternal and child diet	SHS exposure associated with higher child weight status up to 4 years (BMIZ of 0.15 SD; 95% CI: 0.05–0.25) than non-exposed group, p-value not reported
Soesanti et al.^39^ (2019)	Prospective cohort, Indonesia	305	Infant assessed at birth, day 7, and months 1, 2, 4 and 6, postnatally; 46.9% female; 76% exposed to SHS	Self-report by mother (exposed vs not exposed)	WAZ, HAZ, HCZ	Level of education, household income, maternal age and BMI (ΔBMI), parity, and breastfeeding	SHS exposure ≥23 cigarettes/day only associated with lower HC increment (-0.32 mm/m, 95% CI: -0.60, -0.03; p=0.03) than non-exposed group

**Table 2:** Growth measurements in children who had been exposed to SHS in the postnatal period

*Authors (year)*	*Methodology, sample and location*	*N*	*Population/sample characteristics*	*Measurement of SHS exposure*	*Measures of anthropometric functioning*	*Confounders measured*	*Outcomes*
Tielsch et al.^35^ (2009)	A prospective cohort in Tamil Nadu, India	11728	Newborns were followed from birth through 6 months; % female (not given), 39% exposed to SHS	Interview with mothers: exposure to household SHS (reported number of cigarettes smoked in the household per day)	WAZ, HAZ, WHZ	Household demographic and socioeconomic indicators, maternal characteristics, delivery characteristics and the randomized treatment assignments	SHS exposure (1–10 cigarettes/day) not associated with underweight (RR=0.99; 95% CI: 0.93–1.05), stunted (RR=0.94, 95% CI: 0.88–1.02) and wasted (RR=1.02; 95% CI: 0.92–1.12) SHS exposure (≥10 cigarettes/day) also not associated with similar results
Moore et al.^36^ (2017)	Prospective cohort, Colorado, USA	813	Newborns were followed from birth through 5 months; 50% female, 15.9% exposed to SHS	Phone interview with mothers at age 5 months of babies (exposed vs not exposed)	BMIZ, WAZ, WHZ	Maternal: race/ethnicity, education, smoking during pregnancy; household income; Offspring: age, sex, age at introduction of solid foods	SHS exposure not associated with BMI for-age z-score = 0.2 (95% CI: 0.0–0.4; p=0.07) (only among infants who were not exclusively breastfed)
Baheiraei et al.^37^ (2015)	Prospective cohort in southern Tehran, Iran	102	Infant assessed at 3–5 days (baseline), 2 months, and 4 months after birth; 62.7% female; 50% exposed to SHS	Interview with parents (number of cigarettes smoked in the presence of their infants) and infant urinary cotinine	Weight, length and HC	Socio-demographic characteristics, mothers’ cigarette smoke exposure during and after pregnancy and the nutrition condition	SHS exposures associated with lower weight (g) (mean±SD) at two months (exposed: 5258.82±233.6 vs unexposed: 5592.1±216.4; p<0.001) and four months after birth (exposed: 5383.4±272.8 vs unexposed: 5730.3±280.7, p<0.001). Non-exposed infants were taller than the exposed at 4 months after birth (median 60 (60–62) vs 61 (60–62) cm, p<0.001). Head circumference was not significantly different between the two groups at 2 and 4 months of age
Semba et al.^26^ (2007)	Nutritional surveillance system (NSS) in Indonesia	175583	Children 0–59 months of age; 48.0% female; 73.8% exposed to SHS	Interview with parents (exposed vs not exposed)	WHZ, WAZ, HAZ	Age of child; gender; Maternal: age, education, smoking status; Paternal: education, smoking status; Total weekly household expenditure per capita; Number of household members eating from same kitchen	SHS exposure associated with child stunting (OR=1.11; 95% CI: 1.08–1.14, p<0.0001), severe wasting (OR=1.17; 95% CI: 1.03–1.33, p=0.018) and severe stunting (OR=1.09; 95% CI: 1.04–1.15, p<0.001) but not associated with child underweight
Bonu et al.^27^ (2004)	National Family Health Survey-II (NFHS-II) in India	92486	Children aged 0–35 months; % female (not given), 16.1% exposed to SHS	Interview with mothers (exposed vs not exposed)	WAZ, HAZ	Residence (urban/rural), caste, household wealth, and religion at the household level; age and education of mother, and sex of the child at the individual level	SHS exposure associated with severely underweight (OR=1.21; 95% CI: 1.05–1.40; p<0.05) but not associated with severe stunted (OR=1.12; 95% CI: 0.98–1.27)
Best et al.^28^ (2007)	The Bangladesh Nutrition Surveillance Project	77678	Children 0–59 months of age, % female (not given), 69.9% exposed to SHS	Interview with mothers (exposed vs not exposed)	WHZ, WAZ, HAZ	Child age, child gender, maternal age, maternal education level, total monthly household expenditure per capita	SHS exposure associated with an increased risk of stunting (OR=1.17; 95% CI: 1.12–1.21; p<0.0001); underweight (OR=1.17; 95% CI: 1.12–1.22; p<0.0001); wasting (OR=1.10; 95% CI: 1.03–1.17; p=0.004); severe stunting (OR=1.16; 95% CI: 1.10–1.23; p<0.0001), severe underweight (OR=1.21; 95% CI: 1.13–1.30; p<0.0001) and severe wasting (OR=1.142; 95% CI: 0.98 –1.32; p=0.09)
Best et al.^30^ (2008)	The Indonesia Nutrition and Health Surveillance System	438336	Children 0–59 months of age; 46.9% female; 73.7% exposed to SHS	Interview with mother or other adult member of the household (exposed vs not exposed)	WAZ, HAZ	Child age and gender, maternal age, maternal and paternal education, per capita weekly household expenditure and province	SHS exposure was associated with an increased risk of underweight (OR=1.03; 95% CI: 1.01–1.05; p=0.001) and stunting (OR=1.11; 95% CI: 1.09–1.13; p<0.001) and severe underweight (OR=1.06; 95% CI: 1.01–1.10; p=0.020) and severe stunting (OR=1.12; 95% CI: 1.08–1.16; p<0.001)
Chowdhury et al.^31^ (2011)	The Hospital Surveillance System of International Centre for Diarrheal Disease Research, Dhaka Hospital, Bangladesh	13555	Children 0–59 months of age; % female (not given); 49% exposed to SHS	No description (information extracted from a database of hospital-based surveillance system) (exposed vs not exposed)	WHZ, WAZ, HAZ	Child’s age, maternal age, maternal education, family size, socioeconomic status, father’s smoking	Paternal smoking was associated with increased risk of moderate underweight (OR=1.16; 95% CI: 1.08–1.25), severe underweight (OR= 1.15; 95% CI: 1.06–1.26), moderate stunting (OR= 1.15; 95% CI: 1.06–1.23) and severe stunting (OR= 1.13; 95% CI: 1.03–1.25), p-value not reported. Paternal smoking was neither associated with the risk of either moderate or severe wasting
Kyu et al.^32^ (2009)	Cross-sectional DHSs conducted in Cambodia, Dominican Republic, Haiti, Jordan, Moldova, Namibia and Nepal	7289	Children (0–59 months); 48.15% female; 19.7% exposed to SHS	Interview with parents (exposed vs not exposed)	HAZ	Child age and gender, early initiation of breastfeeding within 1 h after birth, mother’s age and education, number of children ever born, child size at birth, household wealth and country of residence	SHS exposure was not associated with stunting (OR=1.004; 95% CI: 0.84–1.19), and severe stunting (OR=1.18; 95% CI: 0.93–1.49)
Raum et al.^21^ (2011)	Cross-sectional study conducted in Aachen, Germany	1954	Children assessed at the age of 6 years; % female (not given); 33.4% exposed to SHS	Interview with parents about exposure during 1st year only, exposure at age 6 years only, exposure at both time periods (exposed vs not exposed)	BMI-for-age percentile	Birth and infancy (birth weight, breast feeding, parity), Children’s current lifestyle factors (watching TV, sports, fast food consumption), Parental factors (education, maternal BMI)	SHS exposure associated with overweight at age 6 years at either one of the two time periods; first year only (OR=2.94; 95% CI: 1.30–6.67), sixth year only (OR=2.57; 95% CI: 1.64–4.04) or at both (OR=4.43; 95% CI: 2.24–8.76), p-value not reported
Yang et al.^38^ (2013)	Cohort of Belarusian children	13889	Children 6.5 years of age; 47.% female; 51.2% exposed to SHS	Self-reported by mother (exposed vs not exposed)	BMI-for-age percentile	Maternal and family characteristics (maternal and paternal age, marital status, number of older children in the household, maternal alcohol consumption during pregnancy, area of residence, and maternal and paternal education, occupation, height, BMI and smoking	SHS exposure associated with higher BMI for maternal smoking (OR=0.2; 95% CI: 0.1–0.3), for paternal smoking (OR=0.1; 95% CI: 0.07–0.2), and increased odds of overweight/obesity for maternal smoking (OR=1.2; 95% CI: 1.0–1.5), for paternal smoking (OR=1.1; 95% CI: 1.0–1.3), p-value not reported
Braithwaite et al.^29^ (2015)	Cross-sectional study (ISAAC Phase Three) in 18 countries)	77192	Children aged 6–7 years; % female (not given); 43.1 % exposed to SHS	Self-reported by parents/guardians, mother smoked in the 1st year of the child’s life and current smoking habits of both parents (exposed vs not exposed)	BMI	Country GNI, centre, individual fast food usage, age and measurement type	SHS exposure associated with greater BMI (+0.11 kg/m^2^; SE=0.04; p=0.002) during first year of life for maternal smoking and greater BMI (maternal smoking: (+0.07; SE=0.03; p=0.03); paternal smoking in high GNI countries: (+0.15; SE=0.02; p<0.0001); but smaller BMI in low GNI countries (−0.14; SE=0.05; p=0.004) in currently smoking parents

From 17 articles selected, the current review explored three anthropometric measurements as the outcome of SHS exposure from each of 8 studies, two anthropometric measurements were captured from each of 2 studies, and only one anthropometric measurement was taken from each of 7 articles. The present review then classified measurements into three groups, comprising weight (weight, WAZ, WHZ and BMI), height (height/length and HAZ), and head circumference.

### SHS exposure and weight of children

Nine studies investigated the effect of SHS exposure on at least one measurement of weight, WAZ or WHZ. While seven studies explored the association between SHS exposure and at least one measurement of BMI (BMI, BMIZ, BMI-for-age percentile or Kaup index). Exposure to SHS was inversely associated with weight outcome (deficit in weight, risk of underweight, risk of wasting) in 7 of 9 studies^26–28,30,31,33,37^. The remaining two studies presented no association between them^35,39^. Only two of nine studies evaluated exposure of SHS during the prenatal period. Furthermore, seven studies conducted in low-income and lower middle-income countries and two other studies performed in upper middle-income countries based on World Bank classification 2019–2020^40^.

SHS exposure was associated with higher BMI or overweight in 4 of 7 studies^17,21,34,38^. One study showed no association^36^ and one study presented an inverse association between SHS exposure and BMI^18^. The last study by Braithwaite et al.^29^ revealed two contrasting results in high and low GNI (gross national income per capita) countries, with SHS exposure associated with higher BMI in high GNI countries and lower BMI in low GNI countries. Three of seven studies, conducted in children aged 6–7 years, found higher BMI in exposed children^21,29,38^. Also, all studies in high-income and upper middle-income countries, except one, were conducted in 18 countries with two levels of GNI.

### SHS exposure and height of children

Ten studies examined the effect of SHS exposure on at least one height indicator (height/length and HAZ), from those two studies conducted in the prenatal period. Eight of ten studies were performed in low-income and lower middle-income countries and the other two were in upper middle-income countries.

SHS exposure was associated with lower length in two studies^33,37^ and a higher risk of stunting in four studies^26,28,30,31^. Nevertheless, exposure to SHS and length/height or risk of stunting were not related in four other studies^27,32,35,39^.

### SHS exposure and head circumference

Three studies evaluated the effect of SHS exposure on the head circumference (HC) in children. Two studies, performed during the prenatal period, found a significant association between SHS exposure and lower HC in children^33,39^. Finally, one study was conducted for the postnatal period, and the head circumference was found not to be significantly different between the exposed and non-exposed to SHS^37^.

## DISCUSSION

This review notes that SHS exposure during the pre or postnatal period has adverse effects on weight and height outcomes in childhood. There is also evidence that SHS exposure in the prenatal period is associated with a lower head circumference. There are several potential mechanisms on how prenatal exposure influences growth in children. SHS contains more than 4000 chemical substances among which are some of the main carcinogenic substances, such as Polycyclic aromatic hydrocarbons (PAHs), 4-aminobiphenyl (ABP), tobacco-specific nitrosamines N’-nitrosonornicotine (NNN), and 4-(methylnitrosamino-) 1-(3,pyridyl)-1-butone (NNK)^41^. PAHs, ABP and N-nitrosamines may cross from the maternal serum to fetus circulation^42-44^. In passive smoker mothers, PAHs and NNK might pass through the placenta and directly influence the children’s hypothalamic centres, which may delay body growth^18^. It is known that the hypothalamus has a vital function in the control of body weight by balancing food intake, energy release, and body fat storage^45^.

Moreover, a study showed that height growth of children exposed to cigarette smoke was lower because the smoke contains cadmium, which disturbs zinc bioavailability^46^. PAHs and NNK may also go through the placenta and directly influence the volume of the fetus anterior cingulate region, and this condition may cause a lower head circumference of the baby^18^. Head growth during prenatal period and infancy is crucial as it is related to subsequent IQ development and is essential in determining how well cognitive abilities are maintained in old age^47,48^.

Another reason might be related to lower nutrition in SHS exposed children, due to family income spent on cigarettes rather than food^26,28,30,49^. Furthermore, SHS causes frequent health problems in infants and children^50^. Based on UNICEF’s conceptual framework on child undernutrition, inadequate dietary intake and frequent illness are immediate causes of child undernutrition^51^. A study by Danaei et al.^52^, in 137 developing countries, demonstrated that fetal growth restriction (FGR) and bad sanitation were the leading risk factors for stunting in developing countries. Passive smoking during pregnancy is notably associated with an increased incidence of FGR. The present review also reveals the association between parental smoking and child stunted growth.

The present review also showed an association between prenatal or postnatal SHS exposure and higher BMI, particularly in children aged 6–7 years. A study by Braun et al.^17^ found stronger effects of tobacco smoke exposure as children become older. Our review is in line with a meta-analysis by Oken et al.^13^ on 14 articles (with 84563 children) and Magalhães et al.^53^ that children whose mothers smoked during the prenatal period were at an elevated risk of becoming overweight in childhood (OR=1.5 and OR=1.43, respectively). A meta-analysis by Qureshi et al.^54^ demonstrated the association between prenatal exposure to environmental tobacco smoke and childhood obesity with OR=1.905.

Prenatal SHS exposure of the mother might cause low birth weight (LBW). It might lead to LBW through the potential pathways of maternal inflammation and lower placental weight^6^. LBW is a proxy-marker of poor fetal growth and nutrition. Based on the Developmental Origins of Health and Disease (DOHaD) hypothesis, the underlying mechanism is poor nutrition (it might be due to nicotine exposure) in utero or during early childhood that affects the risk of disease later in life. Some of the mechanisms begin at the time of the perinatal insult, while other mechanisms perform a more significant part in influencing metabolic disease during the postnatal period (i.e. during catch-up growth). It is similar to the concepts of fetal programming and Barker’s hypothesis, which illustrate the relationship between a specific path of growth—consisting of slow growth in utero and rapidly increasing BMI in postnatal period—and the development of chronic diseases later in life, such as coronary heart disease and related disorders including stroke, hypertension and non-insulin dependent diabetes^55,56^.

Both undernutrition and overnutrition have similar long-lasting physiologic effects. Undernutrition increases susceptibility to fat accumulation, insulin resistance in adulthood, hypertension, dyslipidaemia and a reduced capacity for manual work, among other impairments^57^. Elevated BMI in childhood predicts risk of hypertension in young adulthood, type 2 diabetes, and, to a lesser extent, cardiovascular diseases^58,59^.

### Strengths and limitations

The strengths of this review include its wide-ranging search strategy, systematic data extraction and quality assessment method used. However, there are some limitations. These include the number of participants among extracted articles, the relatively significant difference between study areas, and limited to South-Eastern and Western Asia. These factors might affect the results of the review. At the same time, it reveals the need to further investigate the association between secondhand smoke exposure and growth measurement of children in other countries. Furthermore, the small number of published studies, particularly on head circumference, as an outcome of SHS exposure, prevents us from drawing firm conclusions.

## CONCLUSIONS

The current review emphasizes that growth (below or above the standard) in children may be affected by secondhand smoke exposure pre or postnatally. SHS exposure should thus be considered a modifiable risk factor for underweight, wasting and stunting, specifically in low-income and lower middle-income countries; elevated BMI and overweight particularly in high-income and upper middle-income countries; and small head circumference that might be due to prenatal SHS exposure.

This review implies that it is crucial that people who currently are active smokers, specifically those who live with children or with a pregnant partner, are made aware of the potential effect of tobacco smoke exposure on non-smokers. By encouraging household members to stop smoking (and/or by declining smoking prevalence rates in the population as a whole), the burden of children’s growth problems would also be reduced at the population level. Furthermore, it is also important to encourage families to maintain a smoke-free home environment, and hence education on the health risks of SHS exposure may protect non-smoking women and their children from SHS exposure and its potential negative effects on growth outcomes.

## References

[1] World Health Organizaton Second-hand smoke.

[2] Öberg M, Jaakkola MS, Woodward A, Peruga A, Prüss-Ustün A (2011). Worldwide burden of disease from exposure to second-hand smoke: a retrospective analysis of data from 192 countries. Lancet.

[3] Cheng KW, Chiang WL, Chiang TL (2017). In utero and early childhood exposure to secondhand smoke in Taiwan: A population-based birth cohort study. BMJ Open.

[4] StateUniversity.com Stages of Growth Child Development: Early Childhood (Birth to Eight Years), Middle Childhood (Eight to Twelve Years).

[5] World Health Organization Early child development.

[6] Niu Z, Xie C, Wen X (2016). Potential pathways by which maternal second-hand smoke exposure during pregnancy causes full-term low birth weight. Sci Rep.

[7] Wahabi HA, Mandil AA, Alzeidan RA, Bahnassy AA, Fayed AA (2013). The independent effects of second hand smoke exposure and maternal body mass index on the anthropometric measurements of the newborn. BMC Public Health.

[8] Bachok N, Omar S (2014). The Effect of Second-Hand Smoke Exposure during Pregnancy on the Newborn Weight in Malaysia. Malays J Med Sci.

[9] Mojibyan M, Karimi M, Bidaki R, Rafiee P, Zare A (2013). Exposure to Second-hand Smoke During Pregnancy and Preterm Delivery. Int J High Risk Behav Addict.

[10] Wahabi HA, Alzeidan RA, Fayed AA, Mandil A, Al-shaikh G, Esmaeil SA (2013). Effects of secondhand smoke on the birth weight of term infants and the demographic profile of Saudi exposed women. BMC Public Health.

[11] Leonardi-Bee AJ, Britton J, Venn A (2011). Secondhand Smoke and Adverse Fetal Outcomes in Nonsmoking Pregnant Women: A Meta-analysis. Pediatrics.

[12] Sunday S, Kabir Z (2019). Impact of carers’ smoking status on childhood obesity in the growing up in Ireland cohort study. Int J Environ Res Public Health.

[13] Magalhães EIDS, Sousa BA, Lima NP, Horta BL (2019). Maternal smoking during pregnancy and offspring body mass index and overweight: a systematic review and meta-analysis. Cad Saúde Pública.

[14] Quelhas D, Kompala C, Wittenbrink B (2018). The association between active tobacco use during pregnancy and growth outcomes of children under five years of age: A systematic review and meta-analysis. BMC Public Health.

[15] Salmasi G, Grady R, Jones J, McDonald SD (2010). Environmental tobacco smoke exposure and perinatal outcomes: a systematic review and meta-analyses. Acta Obstet Gynecol Scand.

[16] Ion RC, Wills AK, Bernal AL (2015). Environmental Tobacco Smoke Exposure in Pregnancy is Associated With Earlier Delivery and Reduced Birth Weight. Reprod Sci.

[17] Braun JM, Daniels JL, Poole C (2010). Prenatal environmental tobacco smoke exposure and early childhood body mass index. Paediatr Perinat Epidemiol.

[18] Braimoh TS, Kobayashi S, Sata F (2017). Association of prenatal passive smoking and metabolic gene polymorphisms with child growth from birth to 3 years of age in the Hokkaido Birth Cohort Study on Environment and Children’s Health. Sci Total Environ.

[19] World Health Organization (2010). Nutrition Landscape Information System (NLIS) country profile indicators: interpretation guide.

[20] Ramírez-Vélez R, López-Cifuentes MF, Correa-Bautista JE (2016). Triceps and subscapular skinfold thickness percentiles and cut-offs for overweight and obesity in a population-based sample of schoolchildren and adolescents in Bogota, Colombia. Nutrients.

[21] Raum E, Küpper-Nybelen J, Lamerz A, Hebebrand J, Herpertz-Dahlmann B, Brenner H (2011). Tobacco Smoke Exposure Before, During, and After Pregnancy and Risk of Overweight at Age 6. Obesity.

[22] World Health Organization WHO Child Growth Standards.

[23] Chen R, Clifford A, Lang L, Anstey KJ (2013). Is exposure to secondhand smoke associated with cognitive parameters of children and adolescents? - a systematic literature review. Ann Epidemiol.

[24] Wells GA, Shea B, O’Connell D The Newcastle-Ottawa Scale (NOS) for assessing the quality of nonrandomised studies in meta-analyses.

[25] Takahashi N, Hashizume M (2014). A systematic review of the influence of occupational organophosphate pesticides exposure on neurological impairment. BMJ Open.

[26] Semba RD, Kalm LM, de Pee S, Ricks MO, Sari M, Bloem MW (2007). Paternal smoking is associated with increased risk of child malnutrition among poor urban families in Indonesia. Public Health Nutr.

[27] Bonu S, Rani M, Jha P, Peters DH, Nguyen SN (2004). Household tobacco and alcohol use, and child health: an exploratory study from India. Health Policy.

[28] Best CM, Sun K, de Pee S, Bloem MW, Stallkamp G, Semba RD (2007). Parental tobacco use is associated with increased risk of child malnutrition in Bangladesh. Nutrition.

[29] Braithwaite I, Stewart AW, Hancox RJ, Beasley R, Murphy R, Mitchell EA (2015). Maternal post-natal tobacco use and current parental tobacco use is associated with higher body mass index in children and adolescents: an international cross-sectional study. BMC Pediatr.

[30] Best CM, Sun K, de Pee S, Sari M, Bloem MW, Semba RD (2008). Paternal smoking and increased risk of child malnutrition among families in rural Indonesia. Tob Control.

[31] Chowdhury F, Chisti MJ, Hossain MI, Malek MA, Salam MA, Faruque AS (2011). Association between paternal smoking and nutritional status of under-five children attending Diarrhoeal Hospital, Dhaka, Bangladesh. Acta Paediatr.

[32] Kyu HH, Georgiades K, Boyle MH (2009). Maternal smoking, biofuel smoke exposure and child height-for-age in seven developing countries. Int J Epidemiol.

[33] Fenercioglu AK, Tamer I, Karatekin G, Nuhoglu A (2009). Impaired Postnatal Growth of Infants Prenatally Exposed to Cigarette Smoking. Tohoku J Exp Med.

[34] Robinson O, Mart D, Aurrekoetxea JJ (2016). The Association Between Passive and Active Tobacco Smoke Exposure and Child Weight Status Among Spanish Children. Obesity.

[35] Tielsch JM, Katz J, Thulasiraj RD (2009). Exposure to indoor biomass fuel and tobacco smoke and risk of adverse reproductive outcomes, mortality, respiratory morbidity and growth among newborn infants in south India. Int J Epidemiol.

[36] Moore BF, Sauder KA, Starling AP, Ringham BM, Glueck DH, Dabelea D (2017). Exposure to secondhand smoke, exclusive breastfeeding and infant adiposity at age 5 months in the Healthy Start study. Pediatr Obes.

[37] Baheiraei A, Shamsi A, Mohsenifar A (2015). The effects of secondhand smoke exposure on infant growth: A prospective cohort study. Acta Med Iran.

[38] Yang S, Decker A, Kramer MS (2013). Exposure to parental smoking and child growth and development: A cohort study. BMC Pediatr.

[39] Soesanti F, Uiterwaal CSPM, Grobbee DE, Hendarto A, Dalmeijer GW, Idris NS (2019). Antenatal exposure to second hand smoke of non-smoking mothers and growth rate of their infants. PLoS One.

[40] The World Bank World Bank Country and Lending Groups.

[41] Campbell MA, Ford C, Winstanley MC (2017). What is in secondhand smoke?. Tobacco in Australia.

[42] Zhang X, Li X, Jing Y (2017). Transplacental transfer of polycyclic aromatic hydrocarbons in paired samples of maternal serum, umbilical cord serum, and placenta in Shanghai, China. Environ Pollut.

[43] Annola K, Heikkinen AT, Partanen H, Woodhouse H, Segerbäck D, Vähäkangas K (2009). Transplacental Transfer of Nitrosodimethylamine in Perfused Human Placenta. Placenta.

[44] Myers SR (1996). Characterization of 4-Aminobiphenyl-Hemoglobin Adducts in Maternal and Fetal Blood Samples. J Toxicol Environ Health.

[45] Kim JH, Choi JH (2014). Pathophysiology and clinical characteristics of hypothalamic obesity in children and adolescents. Ann Pediatr Endocrinol Metab.

[46] del Rocio Berlanga M, Salazar G, Garcia C, Hernandez J (2002). Maternal smoking effects on infant growth. Food Nutr Bull.

[47] Gale CR, Walton S, Martyn CN (2003). Foetal and postnatal head growth and risk of cognitive decline in old age. Brain.

[48] Gale CR, O’Callaghan FJ, Bredow M, Martyn CN (2006). The influence of head growth in fetal life, infancy, and childhood on intelligence at the ages of 4 and 8 years. Pediatrics.

[49] Wijaya-Erhardt M (2019). Nutritional status of Indonesian children in low-income households with fathers that smoke. Osong Public Heal Res Perspect.

[50] Centers for Disease Control and Prevention Health Effects of Secondhand Smoke.

[51] United Nations Children’s Fund (2015). UNICEF ’ s approach to scaling up nutrition for mothers and their children.

[52] Danaei G, Andrews KG, Sudfeld CR (2016). Risk Factors for Childhood Stunting in 137 Developing Countries: A Comparative Risk Assessment Analysis at Global, Regional, and Country Levels. PLOS Med.

[53] Oken E, Levitan EB, Gillman MW (2008). Maternal smoking during pregnancy and child overweight: Systematic review and meta-analysis. Int J Obes.

[54] Qureshi R, Jadotte Y, Zha P (2018). The association between prenatal exposure to environmental tobacco smoke and childhood obesity: A systematic review. JBI Database System Rev Implement Rep.

[55] Godfrey KM, Barker DJ (2001). Fetal programming and adult health. Public Health Nutr.

[56] Hoffman DJ, Reynolds RM, Hardy DB (2017). Developmental origins of health and disease: Current knowledge and potential mechanisms. Nutr Rev.

[57] Matrins VJB, Toledo Florêncio TMM, Grillo LP (2011). Long-lasting effects of undernutrition. Int J Environ Res Public Health.

[58] Field AE, Cook NR, Gillman MW (2005). Weight status in childhood as a predictor of becoming overweight or hypertensive in early adulthood. Obes Res.

[59] Park MH, Sovio U, Viner RM, Hardy RJ, Kinra S (2013). Overweight in Childhood, Adolescence and Adulthood and Cardiovascular Risk in Later Life: Pooled Analysis of Three British Birth Cohorts. PLoS One.

