# Supplementary material for: The association between secondhand smoke exposure and growth outcomes of children: A systematic literature review
Source: Tob Induc Dis. 2020 Mar 3;18:12. doi: 10.18332/tid/117958 (PMC7067234; doi:10.18332/tid/117958)
Supplement: Supplementary file 1 [file TID-18-12-s1.pdf]

Appendix A

The Appraisal Standard of Newcastle/Ottawa Scale

Selection

1) Representativeness of the exposed group/cohort
   a) Truly representative of the average parents with their children in the community*
   b) Somewhat representative of the average parents with their children in the community*
   c) Selected group of users (e.g. antenatal care in hospital, volunteers)
   d) No description of the derivation of the group

2) Selection of the non-exposed group/cohort
   a) Drawn from the same community as the exposed group*
   b) Drawn from a different source
   c) No description of the derivation of the non-exposed group

3) Ascertainment of exposure
   a) Secure record (e.g. biomarkers)*
   b) Structured interview or questionnaire*
   c) Written self reports
   d) No description

4) Demonstration that outcome of interest was not present at start of study (Cohort Studies Only)
   a) Yes*
   b) No

Confounder

1) Comparability of groups on the basis of the design or analysis
   a) Study controls for socioeconomic status and education*
   b) Study controls for any additional factor* (e.g.parents age, breastfeeding or food consumption for postnatal SHS, weight and height of parents)

Outcome

1) Assessment of outcome
   a) Independent blind assessment*
   b) Record linkage*
   c) Self reports
   d) No description

2) Was follow-up long enough for outcomes to occur (Cohort Studies Only)
   a) Yes (select an adequate follow up period for outcome of interest)*
   b) No

3) Adequacy of follow up of cohorts (Cohort Studies Only)
   a) Complete follow up – all subjects accounted for*
   b) Subjects lost to follow up unlikely to introduce bias -small number lost- > 70% follow up, or description provided of those lost*
   c) Follow up < 70% and no description of those lost
   d) No statement

There are five items in cross-sectional studies and eight items in cohort studies, respectively. The studies quality was described as follows.

Cross-sectional Studies:

Very Good Studies	: 5 points
Good Studies	: 4 points
Satisfactory Studies	: 3 points
Unsatisfactory Studies	: 0 to 2 points

Cohort Studies :

Very Good Studies	: 7 to 8 points
Good Studies	: 5 to 6 points
Satisfactory	: 4 points
Unsatisfactory Studies	: 0 to 3 points

Appendix B

Table 1. Quality Appraisal (Cross-sectional Studies)

	Semba et al. (2007)	Bonu et al. (2004)	Best et al. (2007)	Best et al. (2008)	Chowdhury et al. (2011)	Kyu et al (2009)	Raum et al. (2011)	Braithwaite et al. (2015)
**Selection**								
1) Representativeness of the exposed group<br>a. Truly representative of the average parents with their children in the community*<br>b. Somewhat representative of the average parents with their children in the community*<br>c. Selected group of users<br>d. No description of the derivation of the group	*	*	*	*	*	*		*
2) Selection of the non-exposed group<br>a. Drawn from the same community as the exposed group*<br>b. Drawn from a different source<br>c. No description of the derivation of the non-exposed group	*	*	*	*	*	*	*	*
3) Ascertainment of exposure<br>a. Secured record (e.g. biomarkers)*<br>b. Structured interview or questionnaire*<br>c. Written self-report<br>d. No description	*	*	*	*		*	*	
**Confounders**								
1) Comparability of groups on the basis of the design or analysis<br>a. Study controls for socioeconomic status and education*<br>b. Study controls for any additional factor (e.g. age, sex, breastfeeding or food consumption for postnatal SHS, weight and height of parents)*	*	*	*	*	*	*	*	*
**Outcome**								
1) Assessment of outcome<br>a. Independent blind assessment*<br>b. Record linkage*<br>c. Self-report<br>d. No description	*		*	*	*	*	*	
**Overall Score**	5/5<br>Very Good	4/5<br>Good	5/5<br>Very good	5/5<br>Very Good	4/5<br>Good	5/5<br>Very good	4/5<br>Good	3/5<br>Satisfactory

Table 2. Quality Appraisal (Cohort Studies)

	Fenercioglu et al. (2009)	Braun et al. (2010)	Braimoh et al. (2017)	Robinson et al. (2016)	Soesanti et al. (2019)	Tielsch et al. (2009)	Moore et al. (2017)	Baheiraei et al. (2015)	Yang et al. (2013)
**Selection**									
1) Representativeness of the exposed Cohort<br>a) Truly representative of the average parents with their children in the community*<br>b) Somewhat representative of the average parents with their children in the community*<br>c) Selected group of users<br>d) No description of the derivation of the cohort			*			*		*	*
2) Selection of the non exposed cohort<br>a) Drawn from the same community as the exposed cohort*<br>b) Drawn from a different source<br>c) No description of the derivation of the non exposed cohort	*	*	*	*	*	*	*		*
3) Ascertainment of exposure<br>a) Secured record (e.g. biomarkers)*<br>b) Structured interview or questionnaire*<br>c) Written self report<br>d) No description		*	*	*		*	*	*	
4) Demonstration that outcome of interest was not present at start of study<br>a) Yes*<br>b) No	*	*	*	*	*	*	*	*	*
**Confounders**									
1) Comparability of groups on the basis of the design or analysis<br>a. Study controls for socioeconomic status and education*<br>b. Study controls for any additional factor (e.g. age,sex, breastfeeding or food consumption for postnatal SHS, weight and height of parents)*	*	*	*	*	*	*	*	*	*

**Outcome**									
1) Assessment of outcome<br>a) Independent blind assessment*<br>b) Record linkage*<br>c) Self report<br>d) No description	*	*	*	*	*	*	*	*	*
2) Was follow-up long enough for outcomes to occur<br>a) Yes (select adequate follow up period for outcome of interest)*<br>b) No	*	*	*	*	*	*	*		*
3) Adequacy of follow up of cohorts<br>a) Complete follow up -all subjects accounted for*<br>b) Subjects lost to follow up unlikely to introduce bias -small number lost- >70% follow up, or description provided of those lost*<br>c) Follow up rate <70% and no description of those lost<br>d) No statement	*			*	*	*	*	*	*
**Overall Score**	6/8<br>Good	6/8<br>Good	7/8<br>Very good	7/8<br>Very good	6/8<br>Good study	8/8<br>Very good	7/8<br>Very good	6/8<br>Good study	7/8<br>Very good

© 2020 Nadhiroh S.R. et al.
